# Identification of the nuclear localisation signal of *O*-GlcNAc transferase and its nuclear import regulation

**DOI:** 10.1038/srep34614

**Published:** 2016-10-07

**Authors:** Hyeon Gyu Seo, Han Byeol Kim, Min Jueng Kang, Joo Hwan Ryum, Eugene C. Yi, Jin Won Cho

**Affiliations:** 1 Department of Integrated OMICS for Biomedical Science, Graduate School, Yonsei University, 50 Yonsei-ro, Seodaemun-gu, Seoul 03722, Republic of Korea; 2 Department of Molecular Medicine and Biopharmaceutical Sciences, School of Convergence Science and Technology and College of Medicine or College of Pharmacy, Seoul National University, 28 Yeongeon-dong, Jongno-gu, Seoul 03080, Republic of Korea

## Abstract

Nucleocytoplasmic *O*-GlcNAc transferase (OGT) attaches a single GlcNAc to hydroxyl groups of serine and threonine residues. Although the cellular localisation of OGT is important to regulate a variety of cellular processes, the molecular mechanisms regulating the nuclear localisation of OGT is unclear. Here, we characterised three amino acids (DFP; residues 451–453) as the nuclear localisation signal of OGT and demonstrated that this motif mediated the nuclear import of non-diffusible β-galactosidase. OGT bound the importin α5 protein, and this association was abolished when the DFP motif of OGT was mutated or deleted. We also revealed that *O*-GlcNAcylation of Ser389, which resides in the tetratricopeptide repeats, plays an important role in the nuclear localisation of OGT. Our findings may explain how OGT, which possesses a NLS, exists in the nucleus and cytosol simultaneously.

*O*-linked-N-acetylglucosamine (*O*-GlcNAc) modification occurs on serine or threonine residues of various proteins in the nucleus and cytoplasm, similar to phosphorylation^1^. Since the discovery of *O*-GlcNAcylation by Hart and Torres in 1984^2^, *O*-GlcNAc has been implicated in many fundamental biological processes. These processes include various signalling pathways, proteasomal degradation, epigenetic regulation, protein-protein interactions, transcription, translation and the stress response^3^,^4^,^5^,^6^,^7^,^8^. *O*-GlcNAcylation is reversible and highly dynamic, and is controlled by only two enzymes. *O*-GlcNAc transferase (OGT) catalyses the addition of *O*-GlcNAc and *O*-GlcNAcase removes *O*-GlcNAc^9^,^10^. *O*-GlcNAc modification interplays with phosphorylation in a reciprocal and competitive manner^11^. The gene encoding OGT was first described in rat liver and is ubiquitously expressed in higher eukaryotes^12^. In mammals, the three variants of OGT are synthesised by alternative splicing of the amino-terminus tetratricopeptide repeat (TPR) domain. Nucleocytoplasmic OGT contains 13.5 domains and is found in the nucleus and cytoplasm. mOGT, the mitochondrial OGT, has a mitochondrial targeting sequence with nine TPRs, and sOGT, the short form OGT, only contains three TPRs^13^,^14^,^15^. The crystal structure of the TPR domain of human OGT is similar to the transport protein importin α and shows the enzyme as a dimer with a large super helix at the inner surface^16^. OGT is also comprised of a C-terminal catalytic domain, whose crystal structure indicates there is a pivot point between the twelfth and thirteenth TPR, a flexible region called a hinge that is capable of large motions^17^. Although there are reports of the subcellular translocation of OGT^18^,^19^, the mechanisms underlying how OGT can be both localised in the nucleus and remain in the cytoplasm is obscure.

The transport of proteins between the nucleus and cytoplasm is a highly regulated process. Nuclear import substrates possess nuclear localisation signals (NLS), which are recognised by distinct transport factors such as importin αs^20^,^21^,^22^,^23^,^24^,^25^,^26^,^27^. Importin αs act as adaptors by binding to both the import substrate and importin β. This trimeric import complex docks to the nuclear pore complex through importin β and translocates into the nucleus^28^,^29^,^30^. The mono- or bipartite motif of NLS is recognised by different members of the importin α family, which is divided into α1, α3, α4, α5, α6 and α7. On the other hand, only one importin β has been described in humans^23^,^24^,^25^,^31^,^32^,^33^,^34^. Although nuclear import via the canonical mechanism is most common, other proteins enter the nucleus independently because of their ability to interact directly with components of nuclear pore complexes^35^.

Here, we identified a sequence of three amino acids (DFP) in OGT that act as a NLS. Moreover, we revealed that nuclear import of OGT is mediated by importin α5. We also elucidated that *O*-GlcNAcylation of the TPR domain of OGT is required for its direct nuclear translocation. Overall, our data suggest that both the NLS and *O*-GlcNAc modification of OGT are required for its nuclear localisation.

## Results

### The DFP motif plays a role in the nuclear localisation of OGT

**Figure 1:**
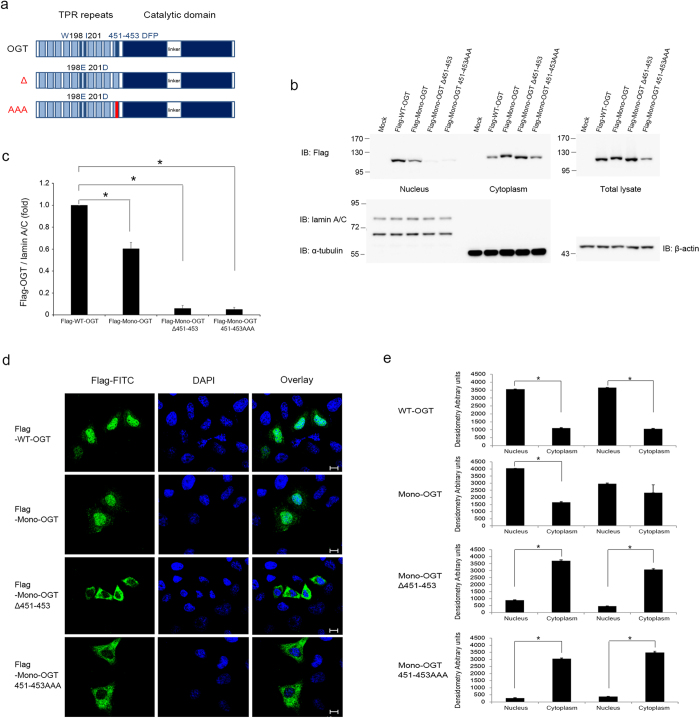
Identification of the NLS of OGT. (**a**) Schematic representation of the OGT constructs used. In Mono-OGT, Trp198 and Ile201 were mutated to glutamate and aspartate, respectively. Δ, deleted DFP motif; AAA, substituted DFP motif. (**b**) WT-OGT and various OGT mutants were expressed as Flag-tagged proteins in HeLa cells grown in 10 cm plates. Subcellular fractionation was performed and aliquots of the fractions were analysed by western blotting with an α-Flag antibody to detect OGT, and with α-lamin A/C, α-α-tubulin and α-β-actin antibodies as markers of the nuclear, cytoplasmic and total fractions, respectively. Images of western blot immunoblotted with an α-Flag antibody, was stripped, and then re-immunoblotted with α-lamin A/C, α-α-tubulin and α-β-actin antibodies respectively. Full gel blots for the cropped blots (**b**) are in the supplementary Fig. 7. (**c**) The band intensities of nuclear imported Flag-OGT in (**b**) were quantified by densitometry and normalised to the laminA/C band intensity. *P < 0.01 (Student’s *t*-test), mean ± s.d. Full gel blots for the statistics (**b**) are in the supplementary Fig. 8. (**d**) HeLa cells were grown on coverslips and transfected with Flag-tagged WT-OGT, Mono-OGT, Mono-OGT Δ451–453 or Mono-OGT 451–453 AAA. Cells were stained with an α-Flag antibody (green) and then analysed by fluorescence microscopy. Nuclei were stained with DAPI (blue). Scale bar, 10 μm. (**e**) Densitometry readings of five separate locations within the nucleus were averaged and this was compared with the mean measurement in five separate locations within the cytoplasm of each cell. Data were quantified using MetaMorph software. Data show mean ± s.d.; n = 5 locations in the cell. *P < 0.01 (Student’s *t*-test). All data are representative of at least three independent experiments.

To identify the NLS of OGT, we generated deletion mutants of OGT and determined their subcellular localisation. HeLa cells were transfected with OGT fused to GFP at the N-terminus and subjected to subcellular fractionation. The first deletion mutant (residues 1–453) contained 13 TPRs, including the sixth and seventh repeats (the local dyad axis of the homodimer)^16^, and localised in the nucleus and cytoplasm. The second deletion mutant lacked the N-terminal 453 amino acids (residues 454–1036) and localised exclusively in the cytoplasm. The third construct contained three more amino acids downstream of the thirteenth TPR (residues 451–1036) and was detected in the nucleus and cytoplasm (Supplementary Fig. 1). Taken together, these data suggest that the residues 451–453 are important for the nuclear import of OGT. To further demonstrate that these three amino acids are the NLS of OGT, we generated Mono-OGT, which excluded the possibility of an interaction between mutant OGT and endogenous OGT, because the tendency of homodimerisation of OGT is decreased when two hydrophobic residues are replaced with negatively charged residues (W198E and I201D)^16^. The interaction between Mono-OGT and endogenous OGT was significantly decreased compared to WT-OGT (Supplementary Fig. 2). Furthermore, the three amino acids of interest (Asp451, Phe452 and Pro453) were mutated to alanine (451–453 AAA) or deleted (Δ451–453; Fig. 1a). Both Mono-OGT 451–453 AAA and Mono-OGT Δ451–453 were distributed largely in the cytoplasm (Fig. 1b–e). Taken together, these results indicate that we identified the NLS of OGT that plays a role in localising OGT to the nucleus.

### The DFP motif can function as a NLS independently

**Figure 2:**
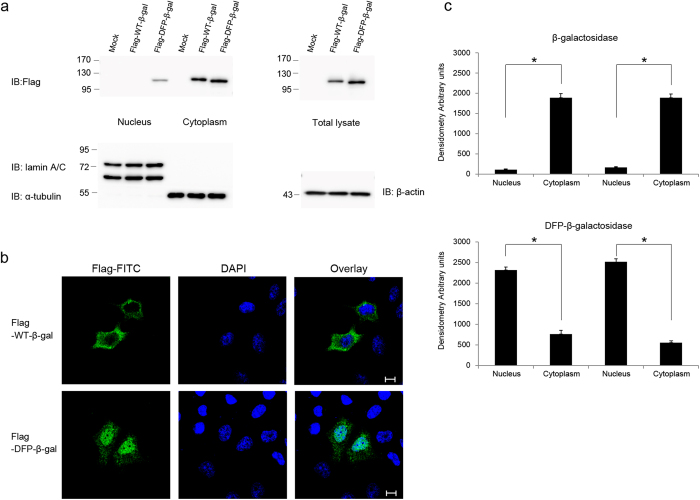
The DFP motif is an independent NLS. (**a**) Subcellular fractionation was performed of HeLa cells transfected with Flag-tagged WT-β-galactosidase or DFP-fused β-galactosidase. Western blotting of aliquots of the fractions was performed with an α-Flag antibody to detect the β-galactosidase proteins, and with α-lamin A/C, α-α-tubulin and α-β-actin antibodies as markers of the nuclear, cytoplasmic and total fractions, respectively. Images of western blot immunoblotted with an α-Flag antibody, was stripped, and then re-immunoblotted with α-lamin A/C, α-α-tubulin and α-β-actin antibodies respectively. Full gel blots for the cropped blots (**a**) are in the supplementary Fig. 7. (**b**) Immunofluorescence confirmed the subcellular fractionation results. HeLa cells transiently overexpressing Flag-tagged β-galactosidase constructs were fixed and stained with both an α-Flag antibody (green) and DAPI (blue). Scale bar, 10 μm. (**c**) The mean of densitometry readings in five separate locations within the nucleus was obtained and this was compared with the mean measurement of five separate locations within the cytoplasm of each cell. Data were quantified using MetaMorph software. Data show mean ± s.d.; n = 5 locations in the cell. *P < 0.01 (Student’s *t*-test). All data are representative of at least three independent experiments.

To validate the importance of the DFP motif of OGT and to examine whether it can act as a NLS independently, we used Flag-tagged β-galactosidase. This protein was too big to freely diffuse into the nucleus and localised exclusively in the cytoplasm when expressed in HeLa cells. However, the addition of DFP to the N-terminus of Flag-tagged β-galactosidase induced its nuclear translocation (Fig. 2a–c), and this was prevented by mutation of DFP to AFP, DAP, DFA or AAA (Supplementary Fig. 4a). Then we wanted to test whether the putative NLS of OGT reported in 1997^13^ has an actual function as nuclear import. However, fusion of putative NLS (residues 477–493) to the Flag-tagged β-galactosidase did not cause import into the nucleus (Supplementary Fig. 4b). We also used Flag tagged double-stranded RNA-specific editase 1 (ADARB1) to test the function of DFP motif in ADARB1. ADARB1 mutated DFP (residues 171–173) to AAA showed greatly reduced nuclear import than WT-ADARB1 (Supplementary Fig. 6). ADARB1 was still localised in the nucleus because ADARB1 interacts with other endogenous ADARB1 in the cells. These results clearly demonstrate that the DFP motif functions as nuclear import independently.

### Importin α5 interacts with OGT

**Figure 3:**
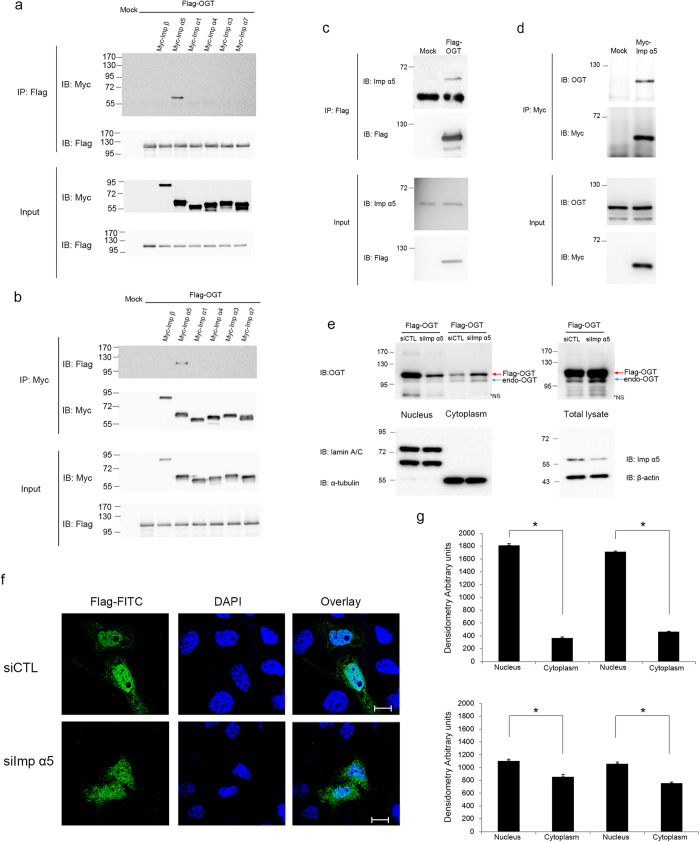
Binding of importin proteins to OGT. (**a**) HeLa cells were co-transfected with Flag-tagged OGT and Myc-tagged importin α or β. Cell lysates were immunoprecipitated with an α-Flag antibody. Co-immunoprecipitated importin α or β, as well as the loading amounts, were analysed by western blotting with an α-Myc antibody. An α-Flag antibody immunoblotting confirmed that equal amounts of OGT constructs were immunoprecipitated. (**b**) Cells were transfected as in (**b**) were immunoprecipitated with an α-Myc antibody. Co-immunoprecipitated OGT was blotted with an α-Flag antibody. An α-Myc antibody immunoblotting confirmed that equal amounts of importin α and β were immunoprecipitated. (**c**) HeLa cells were transfected with Flag-tagged OGT and immunoprecipitated with an α-Flag antibody. Bound endogenous importin α5 was detected by an α-importin α5 antibody. Total lysates were blotted with an α-importin α5 antibody as a loading control. (**d**) HeLa cells transiently overexpressing Myc-tagged importin α5 were immunoprecipitated with an α-Myc antibody. Co-immunoprecipitated endogenous OGT was detected by an α-OGT antibody. Total lysates were blotted with an α-OGT antibody to monitor the amount of OGT. (**e**) HeLa cells were transfected twice with siRNA targeting importin α5 or control siRNA. After 3 days, cells were transfected with Flag-tagged OGT. After another day, cells were subjected to subcellular fractionation. Western blotting was performed on the cytoplasmic and nuclear fractions. Total lysates were blotted with an α-importin α5 antibody to monitor the reduction in endogenous importin α5 and with an α-β-actin antibody as a loading control. *NS; non-specific. **(a–e)** Full gel blots (**a**–**e**) are in the supplementary Fig. 7. (**f**) Immunofluorescence confirmed the subcellular fractionation results. Cells were prepared as described in (**e**), fixed, stained with an α-Flag antibody (green) and DAPI (blue). Scale bar, 10 μm. (**g**) The mean of densitometry readings in five separate locations within the nucleus was obtained and was compared with the mean readings of five separate locations within the cytoplasm of each cell. Data were quantified using MetaMorph software. Data show mean ± s.d.; n = 5 locations in the cell. *P < 0.01 (Student’s *t*-test). All data are representative of at least three independent experiments.

Because nuclear transport of proteins commonly requires their interaction with importin αs^34^,^36^, we determined whether OGT associates with importin αs. The classical nucleocytoplasmic import pathway is mediated by the importin α/β heterodimer^28^,^29^. While only one importin β isoform exists, six human importin αs have been reported^37^. Although the importin αs differ in terms of their cell- and tissue-specific expression patterns, most are expressed ubiquitously, except for importin α6, which is only present in testes^24^. Therefore, we tested the association of OGT with the other five importin αs. No binding was observed between OGT and importin β, α1, α3, α4, or α7 (Fig. 3a,b). However, overexpressed OGT interacted with overexpressed or endogenous importin α5 (Fig. 3a–d). To determine whether importin α5 is also required for the nuclear localisation of OGT, we depleted importin α5 in HeLa cells by RNA interference (RNAi). In cells treated with small interfering RNA (siRNA) targeting importin α5, the amounts of overexpressed OGT localised in the nucleus and cytosol were decreased and increased, respectively (Fig. 3e–g). Thus, our results suggest that importin α5 is an important karyopherin of OGT.

### The DFP motif is required for the interaction of OGT with importin α5

**Figure 4:**
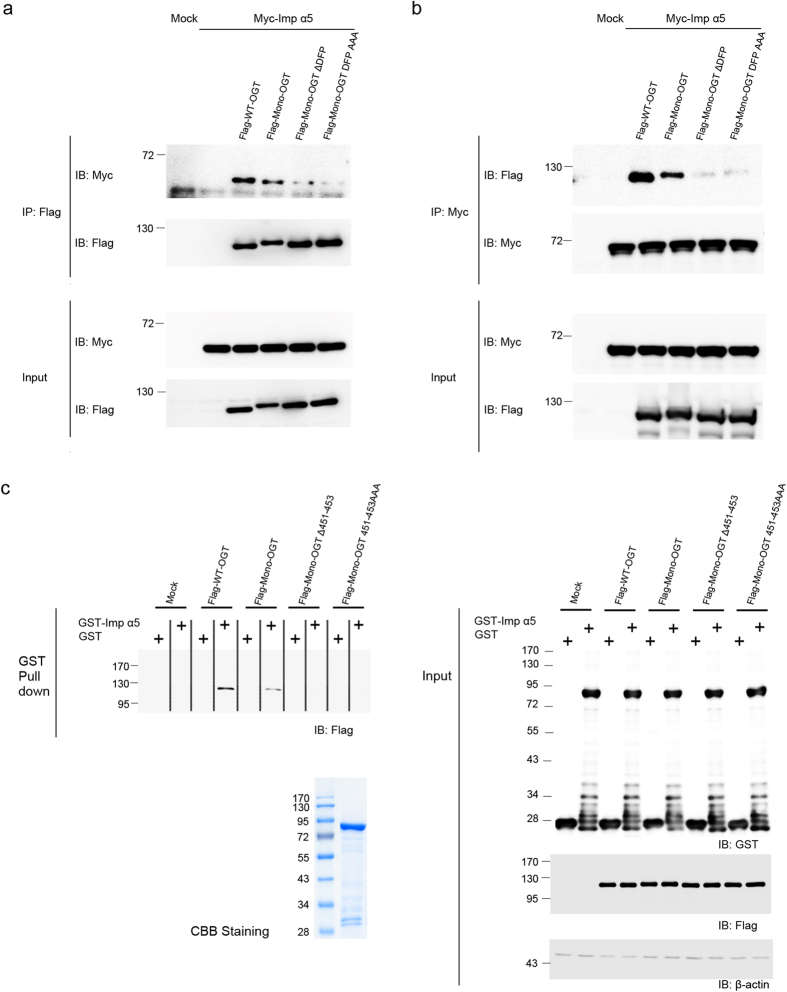
The DFP motif of OGT interacts with importin α5. (**a**) HeLa cells transiently overexpressing Flag-tagged WT-OGT, Mono-OGT, Mono-OGT Δ451–453 or Mono-OGT 451–453 AAA together with Myc-tagged importin α5 were immunoprecipitated with an α-Myc antibody and the beads were stringently washed three times. Co-immunoprecipitated OGT constructs were detected by an α-Flag antibody. Blotting with an α-Myc antibody revealed that equal amounts of importin α5 were immunoprecipitated. (**b**) HeLa cells transfected with Flag-tagged WT-OGT, Mono-OGT, Mono-OGT Δ451–453 or Mono-OGT 451–453 AAA together with Myc-tagged importin α5 were immunoprecipitated with an α-Flag antibody. Following stringent washes, co-immunoprecipitated importin α5 was detected by an α-Myc antibody. Blotting with an α-Flag antibody revealed that equal amounts of the OGT constructs were immunoprecipitated. (**c**) HeLa cells were transfected with Flag-tagged WT-OGT, Mono-OGT, Mono-OGT Δ451–453 or Mono-OGT 451–453 AAA. Cell lysates were incubated with immobilised recombinant GST-importin α5. GST-importin α5 was precipitated, and the associated OGT constructs were detected by western blotting using an α-Flag antibody. GST-importin α5 was detected by Coomassie staining to verify the amount of protein used in the assay. (**a**–**c**) Full gel blots for the cropped blots (**a**–**c**) are in the supplementary Fig. 7. All data are representative of at least three independent experiments.

The finding that importin α5 is involved in the nuclear import of OGT prompted us to examine whether the binding of importin α5 is dependent on the DFP motif of OGT. We first examined the interaction of the various OGT mutants with importin α5. Co-immunoprecipitation experiments revealed that WT-OGT interacted more strongly with importin α5 than Mono-OGT and that Mono-OGT in which the NLS was mutated or deleted showed significantly reduced association with importin α5 (Fig. 4a,b). A slight interaction exists between Mono-OGT Δ451–453, Mono-OGT 451–453 AAA and importin α5 because Mono-OGT still can interact with endogenous OGT (Supplementary Fig. 2). These results were confirmed by *in vitro* binding assays (Fig. 4c). Pulldown experiments were performed in which glutathione S-transferase (GST)-importin α5 fusion protein was incubated with the lysates of HeLa cells transiently overexpressing WT-OGT, Mono-OGT, Mono-OGT Δ451–453 or Mono-OGT 451–453 AAA. No interaction was observed between GST-importin α5 and Mono-OGT Δ451–453 or Mono-OGT 451–453 AAA, as expected. In all experiments, importin α5 interacted more weakly with Mono-OGT than with WT-OGT. This reduced binding affinity of Mono-OGT for importin α5 would explain why the nuclear localisation of Mono-OGT was less extensive than that of WT-OGT (Fig. 1b,c). Taken together, these results indicate that importin α5 has a functional interaction with the NLS of OGT, which affects the nuclear localisation of monomeric OGT.

### *O*-GlcNAcylation of OGT occurs at Ser389

**Figure 5:**
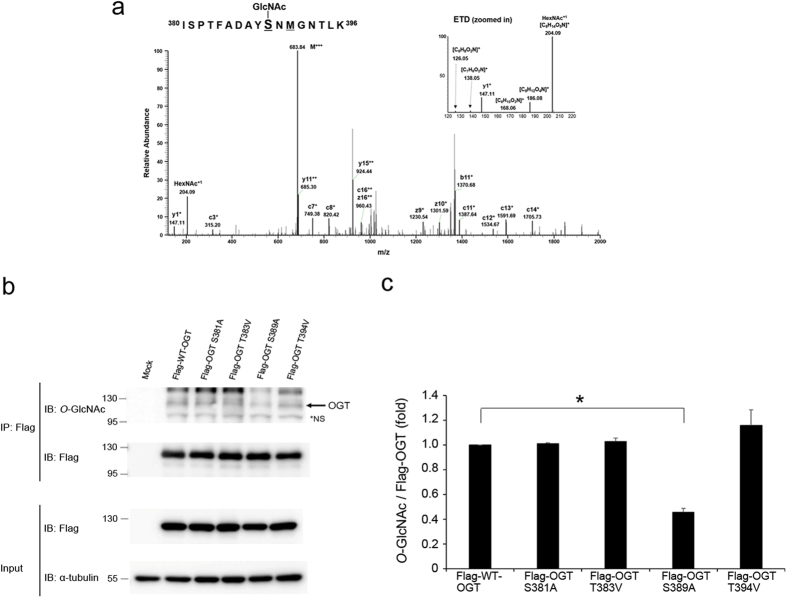
OGT undergoes *O*-GlcNAc modification. (**a**) The ETD MS/MS spectrum of an *O*-GlcNAcylated peptide of OGT1 (residues 380–396) with the triply charged precursor ion m/z 638.9881 (M + 3H)^3+^ is shown. The c- and z-type product ions were assigned. The *O*-GlcNAc oxonium ion (m/z, 204.09) and a series of its fragments (m/z, 186.08, 168.06, 138.05 and 126.05) were also assigned. (**b**) Flag-tagged WT-OGT or OGT point mutants were overexpressed in HEK293 cells. WT-OGT and OGT point mutants were immunoprecipitated with an α-Flag antibody and blotted with an α-*O*-GlcNAc antibody. Blotting with an α-Flag antibody confirmed that equal amounts of the OGT constructs were immunoprecipitated. *NS; non-specific. Full gel blots for the cropped blots (**b**) are in the supplementary Fig. 7. (**c**) The band intensities *O*-GlcNAc in (**b**) were quantified by densitometry and normalised to immunoprecipitated Flag band intensity. *P < 0.01 (Student’s *t*-test), mean ± s.d. All data are representative of at least three independent experiments.

OGT is reportedly modified by *O*-GlcNAc^38^,^39^. However, the sites and functions of *O*-GlcNAc modification of OGT has not been elucidated. To identify the *O*-GlcNAcylated site(s) of OGT, we used HEK293 cells instead of HeLa cells to acquire the necessary amount of Flag-tagged OGT. We separated Flag-tagged OGT immunoprecipitated from HEK293 cell lysates by SDS-PAGE and analysed the protein by mass spectrometry in electron-transfer dissociation (ETD) fragmentation mode. We identified an *O*-GlcNAcylated peptide (amino acids 380–396) of OGT (ISPTFADAY**S**NMGNTLK; Xcorr, 2.808; DeltaCn, 0.477), where the Ser389 residue was modified with *O*-GlcNAc assigned c+ and z+ product ions including distinct *O*-GlcNAc oxonium ions^2^ (m/z 204.09, 186.08, 168.06, 138.05 and 126.05) (Fig. 5a). Next, we created site-specific point mutants of OGT. Mutation of Ser381 and Ser389 with alanine and Thr383, and Thr394 with valine, resulted in a reduction in *O*-GlcNAc modification (Fig. 5b,c). From this investigation, we determined that Ser389 is the major *O*-GlcNAc modification site of OGT. However, we cannot rule out the possibility that other *O*-GlcNAcylation sites exist because OGT was still modified with *O*-GlcNAc, despite mutation of this site.

### *O*-GlcNAc modification of the TPR domain of OGT is important for its nuclear localisation

**Figure 6:**
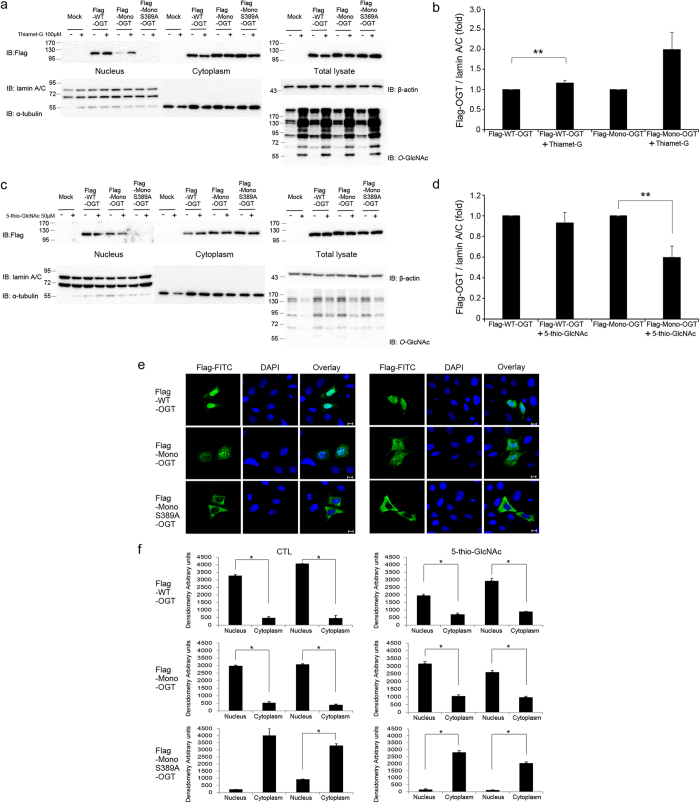
*O*-GlcNAcylation of Ser389 affects the nuclear localisation of OGT. (**a**) HeLa cells were treated with Thiamet-G (100 μM, 4 h) and were then transfected with Flag-tagged WT-OGT, Mono-OGT or Mono-OGT S389A for 24 hours. Cell extracts were subjected to subcellular fractionation. Western blotting of aliquots of the fractions was performed with an α-Flag antibody to detect the OGT constructs, and with α-α-tubulin and α-lamin A/C antibodies as cytoplasmic and nuclear markers, respectively. Images of western blot immunoblotted with an α-Flag antibody, was stripped, and then re-immunoblotted with α-lamin A/C, α-α-tubulin and α-β-actin antibodies respectively. (**b**) The band intensities of nuclear imported Flag-OGT in (**a**) were quantified by densitometry and normalised to the laminA/C band intensity. **P < 0.05 (Student’s *t*-test), mean ± s.d. (**c**) HeLa cells were treated with 5-thio-GlcNAc (50 μM, 4 h) and were then transfected with Flag-tagged WT-OGT, Mono-OGT or Mono-OGT S389A for 24 hours. Cell extracts were subjected to subcellular fractionation as described in (**a**). (**d**) The band intensities of nuclear imported Flag-OGT in (**c**) were quantified by densitometry and normalised to the laminA/C band intensity. **P < 0.05 (Student’s *t*-test), mean ± s.d. (**a**,**c**) Full gel blots for the cropped blots (**a**,**c**) are in the supplementary Fig. 7. (**b**,**d**) Full gel blots for the statistics (**b**,**d**) are in the supplementary Fig. 8. (**e**) Immunofluorescence analysis confirmed the subcellular fractionation results shown in (**b**). HeLa cells were treated with 5-thio-GlcNAc (50 μM, 4 h) or untreated, and then transfected with Flag-tagged WT-OGT, Mono-OGT or Mono-OGT S389A. Thereafter, cells were fixed, stained with an α-Flag antibody (green) and DAPI (blue), and visualised. Scale bar, 10 μm. (**f**) The mean of densitometry readings in five separate locations within the nucleus was obtained and this was compared with the mean measurement of five separate locations within the cytoplasm of each cell. Data were quantified using MetaMorph software. Data show mean ± s.d.; n = 5 locations in the cell. *P < 0.01 (Student’s *t*-test). All data are representative of at least three independent experiments.

**Figure 7:**
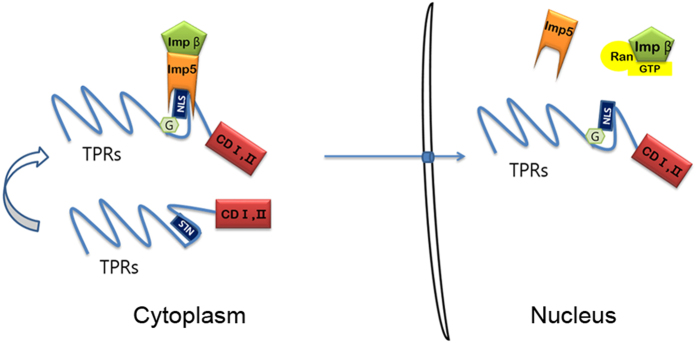
Working model for the nuclear localisation of OGT. In the cytosol, OGT can be *O*-GlcNAcylated on Ser389, which is located in the vicinity of the DFP motif. Without *O*-GlcNAc modification, binding of importin α5 may be hindered because the DFP motif of OGT is hidden. However, once *O*-GlcNAc modification occurs, OGT can interact with importin α5 and hence localise in the nucleus. CD I, II; catalytic domain I, II.

Next, we examined to what extent *O*-GlcNAc modification of OGT contributes to its nuclear localisation. Because Mono-OGT showed less nuclear import than WT-OGT (Fig. 1b–e) and has lower *O*-GlcNAc modification level than that of WT-OGT (Supplementary Fig. 3). Combined with the previous results, we assumed that *O*-GlcNAcylation of OGT may impact its nuclear localisation. To address this issue, we constructed the Mono-OGT S389A mutant. To increase *O*-GlcNAc modification of OGT, cells were treated with Thamet-G, a selective inhibitor of *O*-GlcNAcase^40^. The nuclear localisation of both WT-OGT and Mono-OGT was increased in Thiamet-G-treated cells (Fig. 6a,b). However, the nuclear localisation of Mono-OGT S389A was almost abolished in both Thiamet-G-treated and untreated cells (Fig. 6a). To decrease *O*-GlcNAc modification of OGT, cells were treated with 5-thio-GlcNAc^41^, an inhibitor of OGT. The nuclear localisation of both WT-OGT and Mono-OGT was decreased in 5-thio-GlcNAc-treated cells, and the nuclear localisation of Mono-OGT S389A was completely prevented (Fig. 6c,d). These findings were further supported by fluorescence microscopy analysis (Fig. 6e,f). We predicted that the nuclear localisation of OGT was decreased by a change in its conformation upon exposure of the NLS (residues 451–453). OGT S389A had the same enzyme activity as WT-OGT (Supplementary Fig. 5a,b) and the substitution of Alanine for Ser389 in OGT does not affect protein-protein interaction with other proteins (Supplementary Fig. 5c). This indicated that the decrease of the nuclear localisation of OGT was not due to distortion of its structure, but due to exposure of its NLS. Collectively, our data indicate that the nuclear localisation of OGT is mainly regulated by *O*-GlcNAc modification of Ser389. Our results are summarised in Fig. 7. *O*-GlcNAc modification at Ser389 probably results in exposure of the hidden NLS of OGT and its association with importin α5 and β, resulting in its nuclear localisation.

## Discussion

OGT transfers GlcNAc from uridine diphosphate-N-acetyl glucosamine to the hydroxyl group of a serine or threonine residue on cytoplasmic and nucleus protein substrates^9^,^12^. The TPR domain of the enzyme crystallises as a dimer with an interface between the two subunits^16^. The catalytic domain has a nucleotide-binding domain and there are hinge regions between the twelfth and thirteenth TPRs, where OGT pivots dramatically^17^. Based on these previous studies, we investigated the molecular changes that allow the nuclear import of OGT, which is not fully understood. We identified the NLS of OGT (DFP at position 451–453). Deletion or alanine substitution of DFP abolished the nuclear localisation of OGT and β-galactosidase localised in the nucleus when fused to DFP, suggesting that this NLS plays an essential role in nuclear import. We used Mono-OGT (W198E and I201D)^16^ because endogenous OGT can interact with the exogenously expressed proteins and transport them to the nucleus. Immunoprecipitated Flag-tagged Mono-OGT showed a very weak binding affinity for endogenous OGT (Supplementary Fig. 2), confirming the weak interaction between Mono-OGT and endogenous OGT. This is why very faint bands of Mono-OGT 451–453 AAA and Mono-OGT Δ451–453 were detected in the nuclear fraction (Fig. 1b). Moreover, because Mono-OGT weakly interacted with endogenous OGT, we predicted that the extent of *O*-GlcNAc modification of Mono-OGT is much lower than that of WT-OGT. To test this possibility, we immunoprecipitated Flag from lysates of cells transiently overexpressing Flag-tagged WT-OGT and Mono-OGT. The level of *O*-GlcNAcylated Mono-OGT was considerably lower than that of *O*-GlcNAcylated WT-OGT (Supplementary Fig. 3). This reduced *O*-GlcNAc modification of Mono-OGT decreased its interaction with importin α5 (Fig. 4a–c). Together, these experiments explain why the nuclear importation rate of Mono-OGT was lower than that of WT-OGT. On the other hand, the nuclear localisation of transfected Mono-OGT Δ451–453 and Mono-OGT 451–453 AAA was significantly impaired compared with that of WT-OGT and Mono-OGT (Fig. 1b–e). The identified DFP motif of OGT is not a classical NLS and many proteins that have the same motif localise in the nucleus or remain in the cytoplasm. Surprisingly, the nuclear import of exogenously expressed double-stranded RNA-specific editase 1 (ADARB1) was decreased when its DFP residues at positions 171–173 were mutated to AAA (Supplementary Fig. 6). In these experiments, endogenous ADARB1 was knocked down using RNAi because this protein reportedly forms a homodimer^42^,^43^. Regulation of the nuclear import of other proteins that have a DFP motif should be studied. We postulate that both the NLS and *O*-GlcNAcylation of Ser389 contribute to the nuclear localisation of OGT. *O*-GlcNAc modification might induce a conformational change to facilitate nuclear translocation, similar to other nucleocytoplasmic proteins, such as phosphorylation of extracellular signal-regulated kinase 5 and human telomerase reverse transcriptase^44^,^45^. Additionally, a recent publication showed that phosphorylation of Thr444 of OGT is important for its nuclear localisation^19^. We assume that Thr444 is in close proximity to the DFP motif at positions 451–453 and acts the same as Ser389. In summary, we identified a unique NLS that is responsible for the nuclear localisation of OGT. This NLS controls the nuclear localisation of overexpressed β-galactosidase containing the DFP motif. We also showed that OGT is imported into the nucleus using the DFP motif mediated by importin α5. Our data indicate that *O*-GlcNAcylation of the TPR domain of OGT (Ser389) is required for its nuclear localisation. These findings establish a foundation for how nucleocytoplasmic proteins are regulated and exist in the nucleus and cytosol simultaneously without any other sequestering proteins.

## Online Methods

### Cell culture, DNA transfection and plasmids

HEK293 and HeLa cells were cultured in Dulbecco’s modified Eagle’s medium (Hyclone, Logan, UT) supplemented with 10% foetal bovine serum, 100 U/ml penicillin and 100 μg/ml streptomycin at 37 °C in 5% CO_2_. DNA was transfected using polyethylenimine (Sigma-Aldrich, St Louis, MO) as described previously^46^. siRNA was transfected by lipofection (Lipofectamine Plus; Invitrogen, Carlsbad, CA). Human WT-OGT, mutant OGT, WT-β-galactosidase and mutant β-galactosidase were cloned into the p3xFLAG-CMV™-7.1 Expression Vector (Sigma-Aldrich, St Louis, MO). Human importin proteins were cloned into pRK5 in frame with an N-terminal Myc epitope. Human MEF2C was cloned into pEXPR-IBA105 Strep tag vector (IBA, Goettingen, Germany). The OGT mutants with deletion of residues 451–453 and substitution of residues 451–453 to alanine and β-galactosidase fused to DFP were generated by PCR. The other OGT mutants, including OGT-W198E, I201D (Trp198 mutated to glutamate and Ile201 mutated to aspartate), OGT-S381A (Ser381 mutated to alanine), OGT-T383V (Thr383 mutated to valine), OGT-S389A (Ser389 mutated to alanine) and OGT-T394V (Thr394 mutated to valine), were generated using the QuikChange Site-Directed Mutagenesis Kit (Stratagene, La Jolla, CA). The mutations were confirmed by DNA sequence analysis. To generate GST-tagged importin α5, the cDNA encoding full-length importin α5 was cloned downstream of the GST coding sequence in pGEX-5X (Clontech, Rockville, MD).

### Reagent and antibodies

Thiamet-G was kindly provided by Dr Kwan Soo Kim (Yonsei University, Seoul, Korea) and 5-thio-GlcNAc was kindly provided by David Vocadlo (Simon Fraser University, Canada). Antibodies were used against Flag (F-3156, mouse monoclonal, Sigma-Aldrich, St Louis, MO), Myc (B-14, mouse monoclonal, Santa Cruz, Dallas, Texas), GST (9E10, mouse monoclonal, Santa Cruz), α-tubulin (TU-02, mouse monoclonal, Santa Cruz, Dallas, Texas), β-actin (C-2, mouse monoclonal, Santa Cruz, Dallas, Texas), lamin A/C (#2032, rabbit polyclonal, Cell Signaling, Beverly, MA), MEF2C (#5030, rabbit monoclonal, Cell Signaling, Beverly, MA), OGT (DM17, rabbit polyclonal, Sigma-Aldrich, St Louis, MO) and importin α5 (SAB2500572, goat polyclonal, Sigma-Aldrich, St Louis, MO). CTD110.6, an antibody against *O*-GlcNAc, was purchased from Covance (Princeton, NJ).

### Western blotting, immunoprecipitation and GST precipitation

For western blotting, cells were lysed in NET buffer (150 mM NaCl, 1% Nonidet P-40 [NP-40], 50 mM Tris-HCl and 1 mM EDTA, pH 8.0) supplemented with a protease inhibitor cocktail (Roche, Mannheim, Germany) for 30 min on ice. Protein concentrations were determined by the Bio-Rad protein assay (Hercules, CA). Protein samples were subjected to reducing SDS-PAGE and transferred to nitrocellulose membranes (Amersham, Piscataway, NJ). For immunoprecipitation, cell lysates were gently mixed with specific antibodies and protein A/G beads (Santa Cruz, Dallas, Texas) for 4 h at 4 °C. Immunoprecipitates were washed three times with lysis buffer, eluted with SDS sample buffer and subjected to reducing SDS-PAGE. For co-immunoprecipitation, cells were lysed in co-immunoprecipitation buffer (50 mM Tris-HCl, pH 7.4; 150 mM NaCl; 0.5% NP-40; 1 mM DTT; 0.1 mM EDTA and a protease inhibitor cocktail) and incubated with Flag antibody-conjugated A/G beads for 3 h at 4 °C. Thereafter, the beads were washed three times with co-immunoprecipitation washing buffer (20 mM HEPES, pH 7.4; 2 mM MgCl_2_; 2 mM EGTA; 150 mM NaCl and 0.1% Triton X-100), suspended in sample buffer and subjected to western blotting. Recombinant GST-importin α5 was purified using Glutathione-Sepharose 4B (GE Healthcare), and 5 μg of beads containing bound proteins were incubated with pre-cleared cell lysates for 2 h at 4 °C. The precipitated proteins were washed extensively and subjected to western blot analysis.

### Immunofluorescence microscopy

Cells were grown on precision coverslips (0.17 ± 0.01 mM thickness; Glaswarenfabrik Karl Hecht GmbH & Co KG, Sondheim, Germany) and preparation of the cells were described previously^47^. For immunofluorescence analysis, mouse monoclonal anti-Flag antibodies (1:5000) were applied for 2 h, followed by rinses (2 × 5 min) in PBS containing 1% bovine serum albumin and incubation with the appropriate fluorescent secondary antibodies for 1 h. DAPI was used to stain nuclei. After rinsing, coverslips were mounted on glass slides with Mowiol. Immunofluorescence was recorded with a Zeiss LSM 510 confocal microscope (Zeiss, Jena, Germany) using a Plan-Apochromat 63× objective (1.4 NA). Meta Imaging Series^®^ MetaMorph software (Meta Series Software 7.7.0; Molecular Devices) was used to quantify the data by taking densitometry readings of five separate locations within the nucleus and cytoplasm of each cell.

### SDS-PAGE and in-gel digestion

The eluted OGT sample was loaded onto a 4–12% Bis-Tris NuPAGE gel (NOVEX, San Diego, CA) for electrophoresis and stained with Coomassie Brilliant Blue (Sigma-Aldrich). The gel bands corresponding to OGT were excised and subjected to in-gel tryptic digestion following a general protocol^48^,^49^. Briefly, OGT bands were destained with 50% (v/v) acetonitrile (ACN) prepared in 25 mM ammonium bicarbonate and 100 mM ammonium bicarbonate for 15 min. Proteins were reduced with 20 mM DTT at 60 °C for 1 h and then alkylated with 55 mM iodoacetamide at room temperature for 45 min in the dark. After dehydration, the proteins were digested with 12.5 ng/μl analytical grade porcine trypsin (Thermo Scientific Pierce, Rockford, IL) prepared in 50 mM ammonium bicarbonate overnight at 37 °C. Peptides were then extracted from the gel pieces with 50% (v/v) ACN prepared in 5% formic acid, dried under a Centrivap concentrator (Labconco, Kansas City, MO) and stored at −20 °C until use.

### Mass spectrometry

The peptide samples extracted by in-gel digestion were suspended in 40 μl of solvent A (0.1% formic acid prepared in water, Optima LC/MS grade, Fisher Scientific, Pittsburgh, PA). Thereafter, 2 μl of the sample was loaded onto a house-packed 75 μm (inner diameter of microcapillary) × 15 cm C18 (5 μm, 100 Å) column and separated with a 5–30% gradient of solvent B (0.1% formic acid prepared in ACN) for 90 min at a flow rate of 300 nL/min. Mass spectra were recorded on an Orbitrap Fusion Tribrid mass spectrometer (Thermo Fisher Scientific, San Jose, CA) interfaced with a nanoAcquity UPLC (Waters, Milford, MA). The Orbitrap Fusion Tribrid mass spectrometer was operated in several modes all in Orbitrap, namely, full scan MS1, data-dependent acquisition high-energy collision dissociation scan, product ion-triggered MS3 ETD scan and product ion-triggered MS3 EThcD scan. The raw data were processed using the Trans-Proteomic Pipeline (v4.8.0 PHILAE) and compared with a database composed of human OGT1 (O15294-3, UniProt ID), about 500 decoy proteins and common contaminants. Carbamidomethyl of cysteine was considered the fixed modification, and variable modification was set for oxidation of methionine and *O*-GlcNAcylation of serine and threonine.

### Statistical analysis

Each experiment was repeated three to four times with consistent results. Data are representative of the mean values obtained. Differences between groups were evaluated using the two-tailed unpaired Student’s *t*-test. *P* values < 0.05 were considered to indicate statistical significance for all statistical evaluations. (*P < 0.01 and **P < 0.05).

## Additional Information

**How to cite this article**: Seo, H. G. *et al*. Identification of the nuclear localisation signal of *O*-GlcNAc transferase and its nuclear import regulation. *Sci. Rep.* **6**, 34614; doi: 10.1038/srep34614 (2016).

## Supplementary Material

Supplementary Information
