# Supplementary material for: Identification of the nuclear localisation signal of O-GlcNAc transferase and its nuclear import regulation
Source: Sci Rep. 2016 Oct 7;6:34614. doi: 10.1038/srep34614 (PMC5054401; doi:10.1038/srep34614)
Supplement: Supplementary Information [file srep34614-s1.pdf]

Supplementary information

Identification of the nuclear localisation signal of *O*-GlcNAc transferase and its nuclear import regulation

Hyeon Gyu Seo[1], Han Byeol Kim[1], Min Jueng Kang[2], Joo Hwan Ryum[1],Eugene C. Yi[2] and Jin Won Cho[1,3]

[1]Department of Integrated OMICS for Biomedical Science, Graduate School, Yonsei University, 50 Yonsei-ro, Seodaemun-gu, Seoul 03722, Republic of Korea

[2]Department of Molecular Medicine and Biopharmaceutical Sciences, School of Convergence Science and Technology and College of Medicine or College of Pharmacy, Seoul National University, 28 Yeongeon-dong, Jongno-gu, Seoul 03080, Republic of Korea

[3]Correspondence should be addressed to Jin Won Cho (e-mail: chojw311@yonsei.ac.kr)

Supplementary Figure 1.

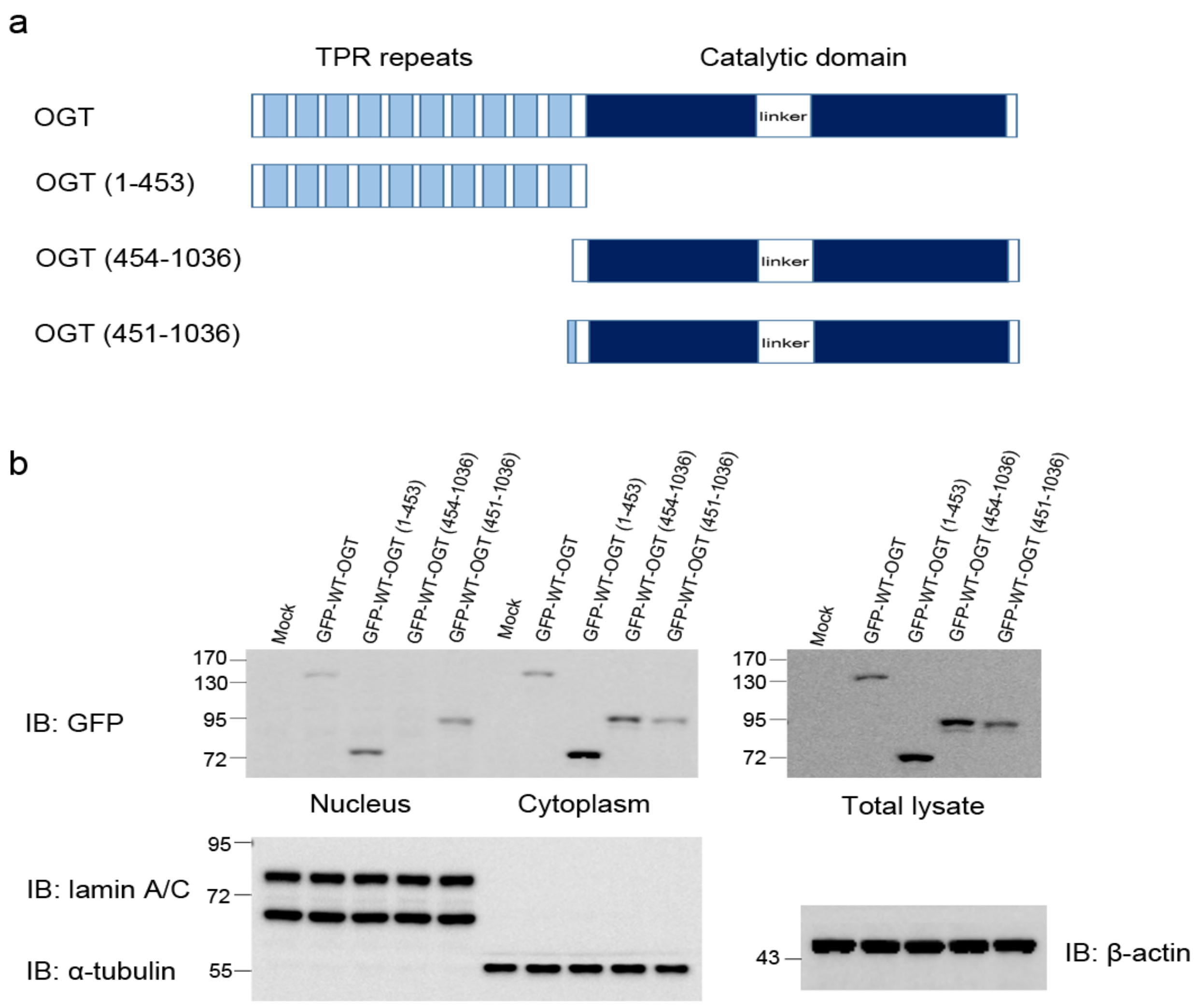

Supplementary Figure 1. Subcellular localisation of OGT deletion mutants

(a) Schematic representation of OGT constructs used. The first deletion mutant (residue 1-453) only contained 13 TPR repeats. The second and third constructs lacked the N-terminal TPR repeats and contained catalytic domain. The third construct contained three more amino acids downstream of the thirteenth TPR (residues 451-1035).

(b) GFP-tagged WT-OGT and various OGT deletion mutants were expressed in HeLa cells. Cells were subjected to subcellular fractionation. Western blotting of aliquots of the fractions was performed with an α-Flag antibody to detect OGT and with α-lamin A/C, α-α-tubulin and α-β-actin as markers of the nuclear, cytoplasmic and total fractions, respectively.

All data are representative of at least three independent experiments.

Supplementary Figure 2.

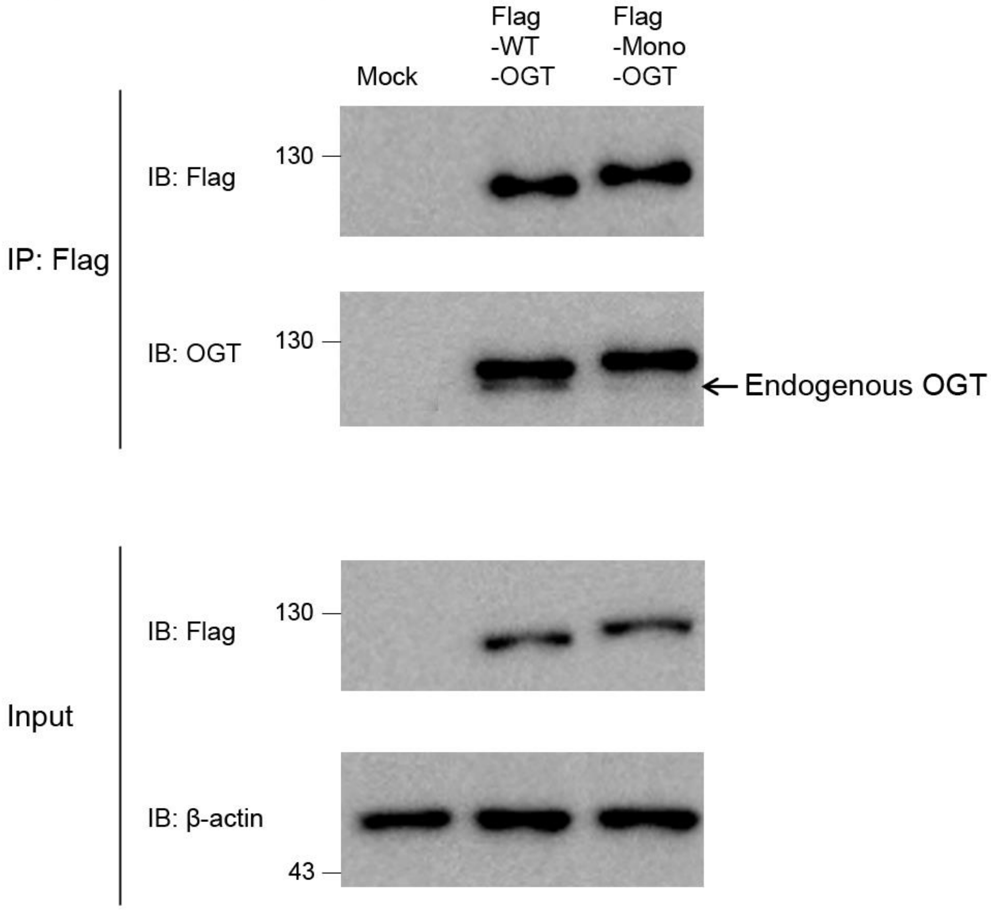

Supplementary Figure 2. Overexpressed OGT interacts with endogenous OGT

Flag-tagged WT-OGT or Mono-OGT (Trp198 and Ile201 mutated to glutamate and aspartate, respectively) was overexpressed in HeLa cells and immunoprecipitated with an α-Flag antibody. Levels of precipitated OGT were determined by an α-Flag antibody, and interacting endogenous OGT was detected by an α-OGT antibody.

All data are representative of at least three independent experiments.

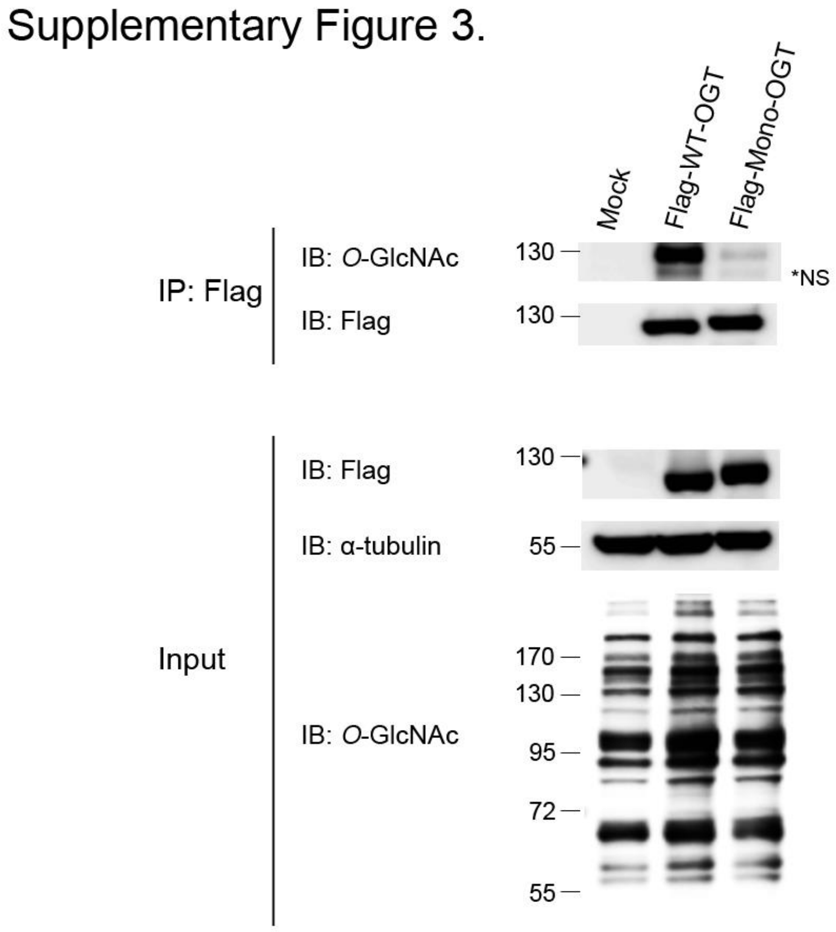

Supplementary Figure 3. Decreased *O*-GlcNAcylation of Mono-OGT

Flag-tagged WT-OGT or Mono-OGT was overexpressed in HeLa cells and immunoprecipitated with an α-Flag antibody. Immunoprecipitates were blotted with an α-*O*-GlcNAc antibody. Blotting with an α-Flag antibody confirmed that equal amounts of OGT were precipitated. *NS; non-specific.

All data are representative of at least three independent experiments.

Supplementary Figure 4.

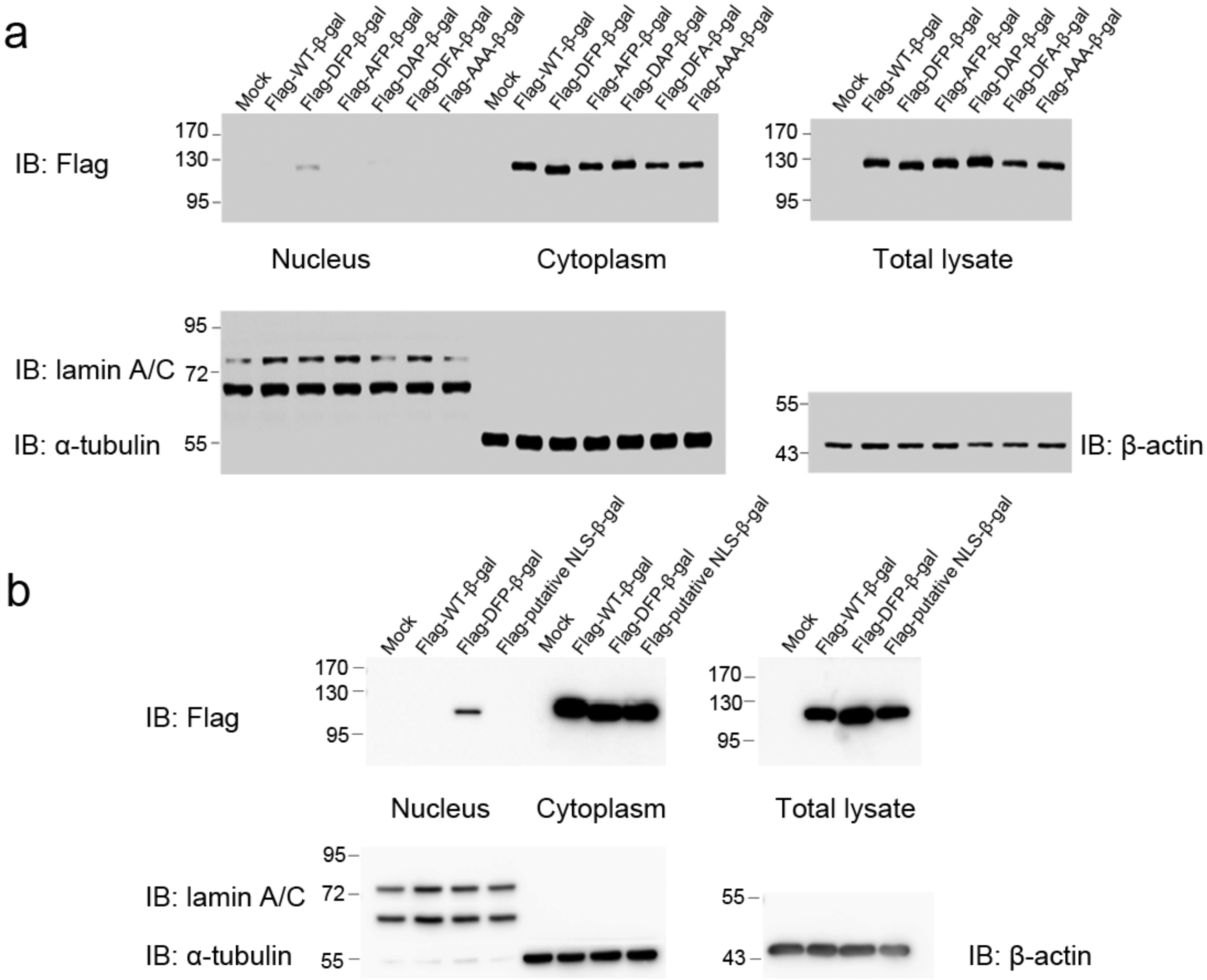

Supplementary Figure 4. The DFP motif can induce β-galactosidase's nuclear import

(a) Subcellular fractionation was performed of HeLa cells transfected with Flag-tagged WT-β-galactosidase, DFP-β-galactosidase, AFP-β-galactosidase, DAP-β-galactosidase, DFA-β-galactosidase or AAA-β-galactosidase. Western blotting of aliquots of the fractions was performed with an α-Flag antibody to detect the -β-galactosidase proteins, and with α-lamin A/C, α-α-tubulin and α-β-actin antibodies as markers of the nuclear, cytoplasmic and total fractions, respectively

(b) HeLa cells were transfected with Flag-tagged WT-β-galactosidase, DFP-β-galactosidase, or putative NLS-β-galactosidase and then subjected to subcellular fractionation as described in (a). DFP; 451-453 residues and putative NLS; 477-493 residues.

All data are representative of at least three independent experiments.

Supplementary figure 5.

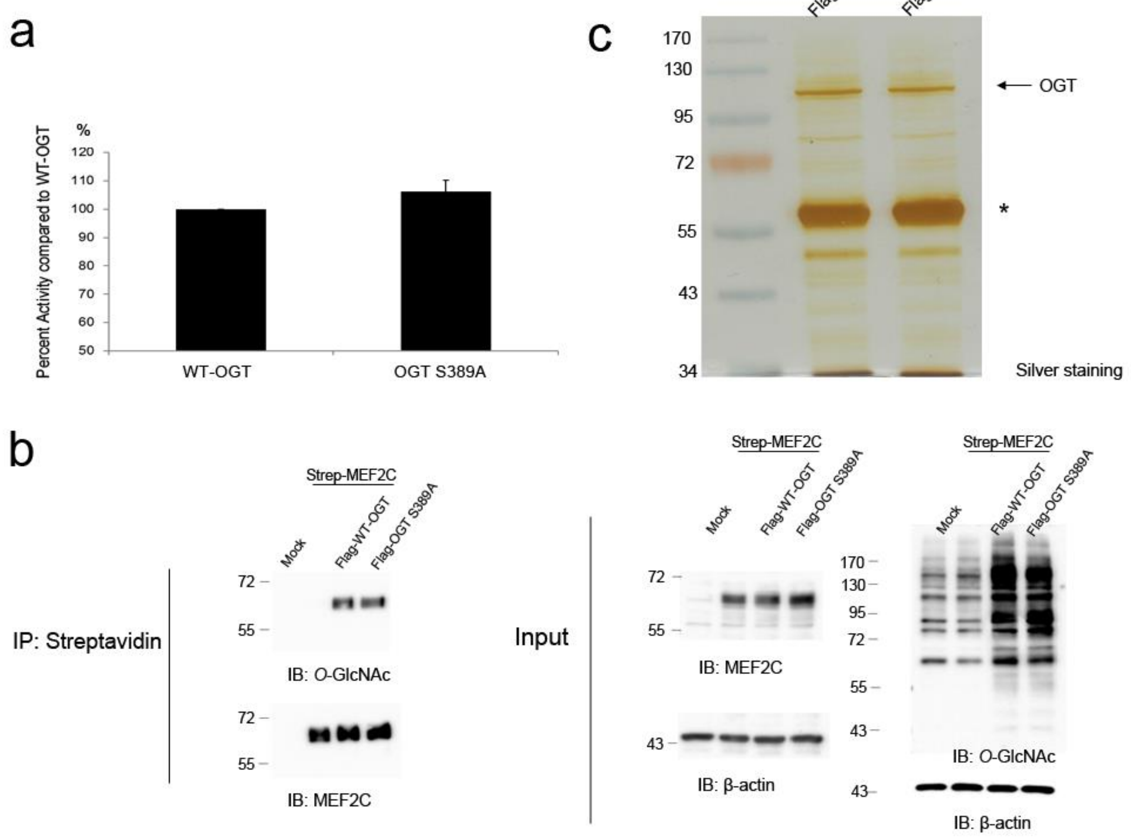

Supplementary Figure 5. The substitution Alanine for Ser389 in OGT does not affect the enzyme activity and protein-protein interaction

(a) Flag-tagged WT-OGT or OGT S389A was expressed in HeLa cells. OGT activity was measured using the casein kinase 2 peptide as a substrate. The y-axis represents activity relative to that of WT-OGT. Data show mean ± s.d.; n=3, data pooled across three independent experiments.

(b) Flag-tagged WT-OGT or OGT S389A were co-expressed with Strep-tagged MEF2C in HeLa cells. Strep-tagged MEF2C was then immunoprecipitated with streptavidin agarose and immunoblotted with for α-*O*-GlcNAc antibody. Blotting with an α-MEF2C antibody confirmed that equal amount of the MEF2C constructs were immunoprecipitated.

(c) HeLa cells were transfected with Flag-tagged WT-OGT or OGT S389A. Cell lysates were immunoprecipitated with an α-Flag antibody and the beads were mildly washed three times. Co-immunoprecipitated proteins were separated by SDS-PAGE and were then stained with silver nitrate. *; IgG heavy chain

All data are representative of at least three independent experiments.

Supplementary Figure 6.

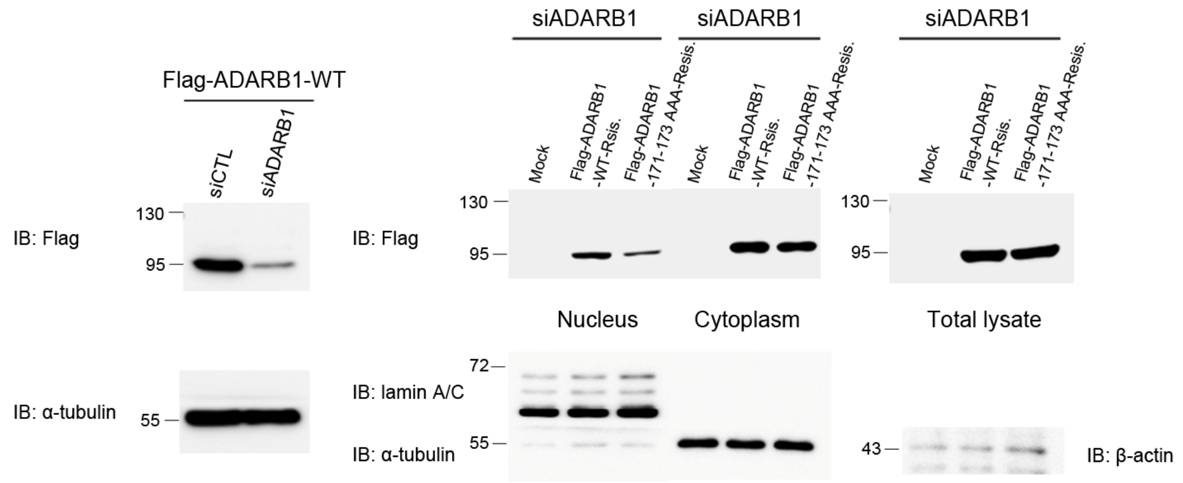

Supplementary Figure 6. The DFP motif mediates the nuclear import of ADARB1

HEK293 cells were transfected with control siRNA or siRNA targeting ADARB1 using Lipofectamine. Forty-eight hours later, cells were transfected with the siRNA-resistant construct of Flag-tagged ADARB1, or ADARB1-171-173 AAA respectively. Subsequently, cells were subjected to subcellular fractionation. Western blotting of aliquots of the fractions was performed with an α-Flag antibody to detect ADARB1 and with α-α-tubulin, α-lamin A/C and α-β-actin antibodies as cytoplasmic, nuclear and total markers, respectively.

All data are representative of at least three independent experiments.

Supplementary Figure 7.

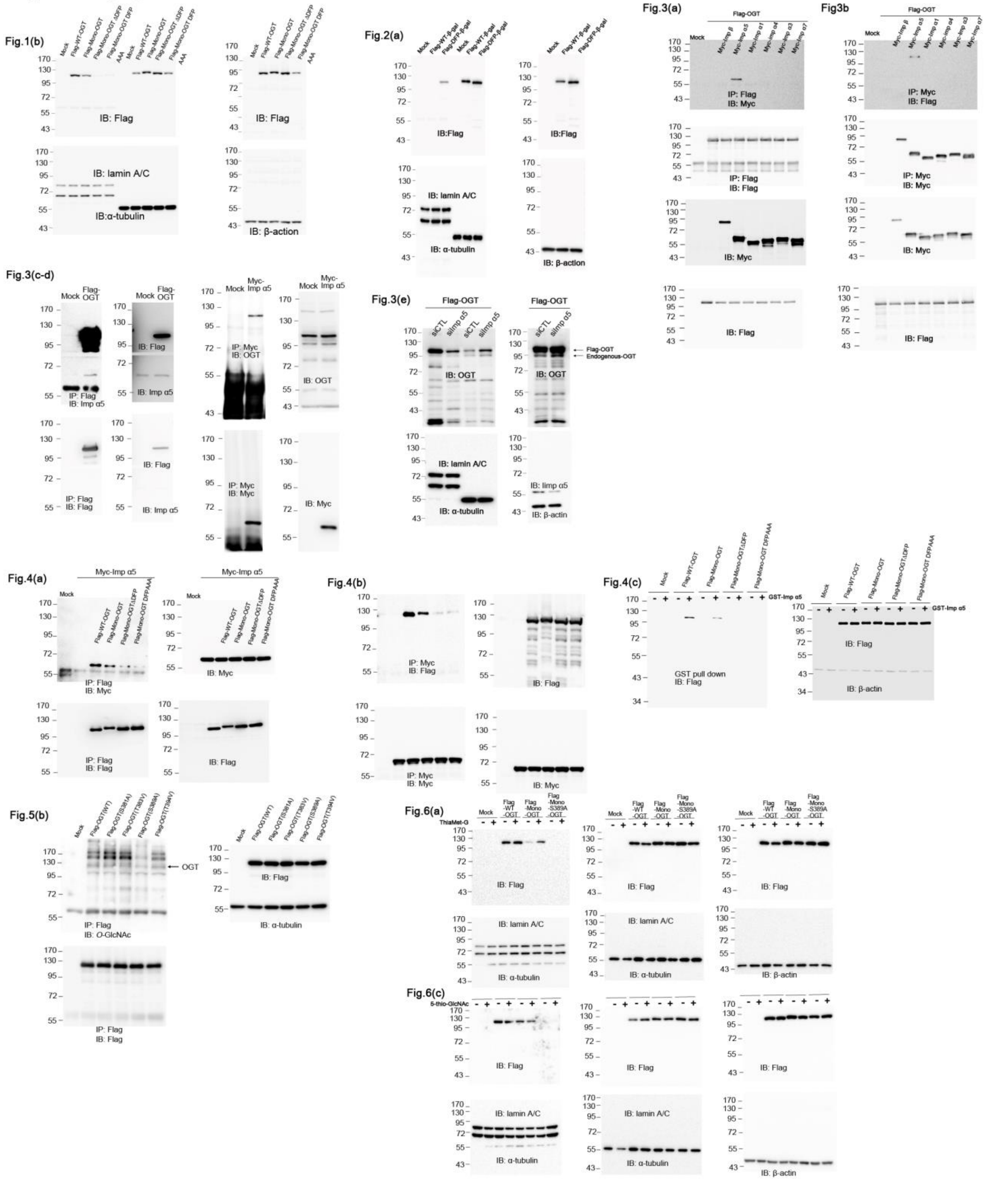

Supplementary Figure 7. Uncropped immunoblots, corresponding to the indicated figures in the manuscript.

Supplementary Figure 8.

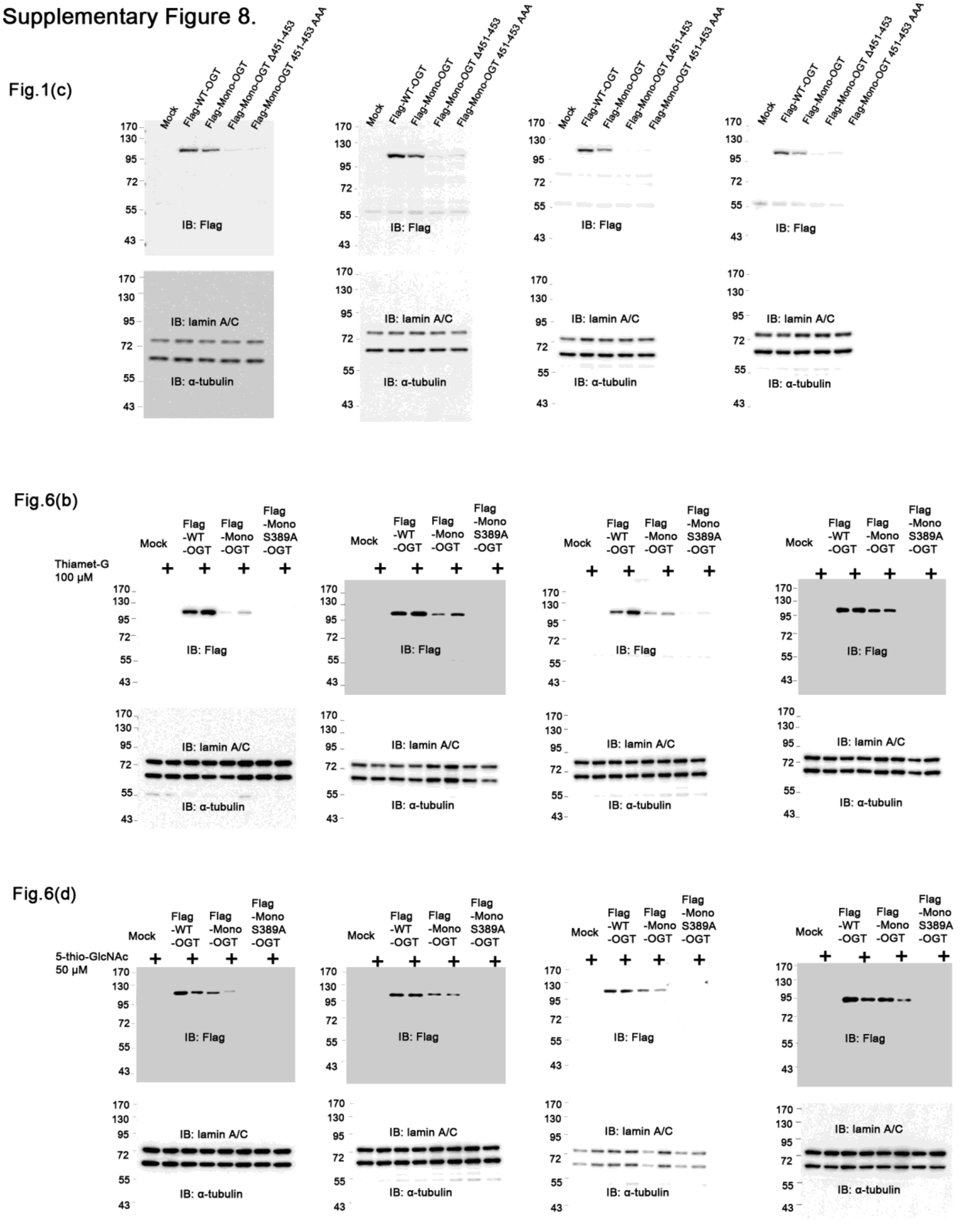

Supplementary Figure 8. Uncropped immunoblots for statistics, corresponding to the indicated figures in the manuscript.

Supplementary methods

Cell fractionation

Transfected HeLa cells were washed with ice-cold PBS, suspended in hypotonic buffer (10 mM HEPES, 10 mM KCl, 0.1 mM EDTA, 0.1 mM EGTA and 1 mM DTT) and vortexed with 0.5% NP-40. The extracts were centrifuged (1500 ×g, 3 min, 4°C). The supernatants, containing the cytosolic fraction, were boiled, and the pellets were washed in hypotonic buffer three times. Thereafter, the pellets were suspended in extraction buffer (20 mM HEPES, 400 mM NaCl, 1 mM EDTA, 1 mM EGTA, 1 mM DTT and 10% glycerol) and centrifuged (12,000 ×g, 10 min, 4°C). The supernatant, containing the nuclear fraction, was subjected to western blotting with appropriate antibodies.

Immunoprecipitation and Western blotting

For immunoprecipitation of Strep-tagged MEF2C, cell lysates were gently mixed with Streptavidin agarose (Merck Millipore, Darmstadt, Germany) for 4 h at 4°C. Immunoprecipitates were washed three times with lysis buffer, eluted with SDS sample buffer and subjected to reducing SDS-PAGE and transferred to nitrocellulose membranes (Amersham, Piscataway, NJ).

siRNA sequences

siRNAs targeting importin α5 were designed as described previously[1]. A scrambled sequence (5’-CCTACGCCACCAATTTCGT-3’) was used as a control and did not correspond to any known gene in the database. The targeting siRNA sequences corresponded to the following coding regions after the start codon: for importin α5, 5’-GGCTCAGATTAGTAACATG-3’ and 5’-AATGTGCTTTCCTGGTTGCTGTT-3’; and for ADARB1, 5’-CGCTCCAATGCGAGCATCCAA-3’.

OGT activity assay

In vitro *O*-GlcNAcylation of the casein kinase 2 peptide PGGSTPVSSANMM was performed as described previously[2,3]. The peptide (1 mM) was incubated with 1 mg of precipitated lysates following a previously described protocol[3].

Supplementary References

1. Quensel, C., Friedrich, B., Sommer, T., Hartmann, E. & Kohler, M. In vivo analysis of importin alpha proteins reveals cellular proliferation inhibition and substrate specificity. *Mol. Cell Biol*. **24**, 10246-10255 (2004).

2. Clarke, A.J. et al. Structural insights into mechanism and specificity of O-GlcNAc transferase. *EMBO J.* **27**, 2780-2788 (2008).

3. Zachara, N.E., Vosseller, K. & Hart, G.W. Detection and analysis of proteins modified by O-linked N-acetylglucosamine. *Curr. Protoc. Mol. Biol.* **Chapter 17**, Unit 17.6 (2011).
